# Non‐ST‐elevated myocardial infarction with “N” wave on electrocardiogram and culprit vessel in left circumflex has a risk equivalent to ST‐elevated myocardial infarction

**DOI:** 10.1002/clc.23334

**Published:** 2020-02-07

**Authors:** Tiangui Yang, Jie Chen, Xiaoxia Liu, Changlu Xu, Tiesheng Niu, Xi Fu, Peng Fu

**Affiliations:** ^1^ Department of Cardiology Shengjing Hospital of China Medical University Shenyang China

**Keywords:** acute non‐ST‐elevated myocardial infarction, acute ST‐elevated myocardial infarction, delayed activation wave, left circumflex

## Abstract

**Background:**

It was found that delayed activation wave often appeared in terminal QRS wave in non‐ST‐elevated myocardial infarction (NSTEMI) with culprit vessel in left circumflex artery (LCX), yet little is known about the similarities among non‐“N”‐wave non‐ST‐elevated myocardial infarction (N‐NSTEMI) and ST‐elevated myocardial infarction (STEMI).

**Hypothesis:**

In AMI patients with the culprit vessel in LCX, “N” wave NSTEMI has a risk equivalent to STEMI.

**Methods:**

All 874 patients admitted to Shenjing Hospital of China Medical University between January 1, 2013 and December 30, 2017 were included and whose coronary angiography (CAG) indicated the culprit vessel in LCX. Patients were divided into three groups: ST‐elevated myocardial infarction group (STEMI group, n = 322), “N” wave non‐ST‐elevated myocardial infarction group (N‐NSTEMI group, n = 232) and non‐“N”‐wave NSTEMI group (non N‐NSTEMI group, n = 320). The basic data and the incidence of MACE during hospitalization and 12 months were analyzed.

**Results:**

In STEMI and N‐NSTEMI groups, AST, CK, CK‐MB, TnI, and stenosis severity were significantly higher than non N‐NSTEMI (*P* < .05). The lesions in the N‐NSTEMI and STEMI groups were more often located proximal LCX before giving rise to OM1 of LCX (*P* < .05), however, the non N‐NSTEMI group was often located distal LCX after giving rise to OM1 and the OM1 (*P* < .05). The incidence rates of all MACEs, all‐cause death, ST, TVR, and rUAP were similar in N‐NSTEMI and STEMI groups, which were greater than non N‐NSTEMI (*P* < .05). Both N‐NSTEMI and STEMI are independent risk factors for MACE (*P* < .05).

**Conclusion:**

The basic data and the incidence of major adverse cardiac event were similar in N‐NSTEMI and STEMI patients, N‐NSTEMI has a risk equivalent to acute STEMI.

## 1 INTRODUCTION

Recently, there has been a shift in the literature towards finding novel electrocardiogram (ECG) changes, which are highly suggestive of total coronary artery occlusion, but lack ST‐elevation in contiguous leads.^1^, ^2^, ^3^ These ST‐elevated myocardial infarction (STEMI)‐equivalents include delayed activation wave (N wave, notch in terminal QRS wave complex II, III, aVF, or I, aVL). In their preliminary study, Niu et al^1^ reported an ECG phenomenon in acute non‐ST‐elevated myocardial infarction (NSTEMI) patients with the culprit vessel in left circumflex artery (LCX): a turning point or notch appeared at the terminal part of the QRS wave complex in leads II, III, and AVF or leads I and AVL, which was called “N” wave. The sensitivity and specificity of “N” wave for detection of circumflex artery lesions were 77% and 96%. The specificity of the “N” wave on the relevant ECG leads detecting acute NSTEMI with LCX culprit vessel was 96%.^1^ However, the role of “N” wave in NSTEMI patients with culprit LCX artery has not yet fully understood. It is not clear if “N” wave can be considered an ST segment elevation equivalent pattern; there is no study to explore the similarities and differences among N‐NSTEMI, non N‐NSTEMI, and STEMI. The ECG manifestations of AMI with culprit vessel in left circumflex artery are not specific, and the clinical features of acute NSTEMI with “N” wave on ECG and coronary angiography with culprit vessel in left circumflex artery remain unclear. Therefore, we analyzed the clinical data of patients with AMI and culprit vessel in left circumflex artery, and explored the similarities and differences between acute NSTEMI with “N” wave in ECG and acute STEMI.

## 2 MATERIAL AND METHODS

### 2.1 Subjects and inclusion criteria

A total of 874 inpatients with acute myocardial infarction (AMI) from January 1, 2013 to December 30, 2017 at Shenjing Hospital of China Medical University, whose CAG revealed the culprit vessel in the LCX were enrolled. Of these, 459 were males and 415 were females. Written informed consents were obtained from all enrolled AMI inpatients. The study was approved by the Ethics Committee of Shengjing Hospital, and was performed in accordance with the Declaration of Helsinki principles. All patients were underwent percutaneous coronary intervention (PCI). The time‐to‐operation (door‐to‐balloon time), location and degree of stenosis of the affected vessel, and intervention information were recorded. Myocardial infarction markers test time: at the first 3 days, they were tested once every 4 to 12 hours, including admission immediate, pre‐PCI and post‐PCI, they were tested once a day 3 days later, and record the results of all myocardial infarction markers and find the highest value. In addition, the occurrence of complications during PCI and major adverse cardiac events (MACEs) during hospitalization and 12 months were recorded,^3^, ^4^ including cardiogenic shock, no‐reflow, stent thrombosis (ST), target vessel revascularization (TVR), target lesion revascularization (TLR), recurrent unstable angina pectoris (rUAP), all‐cause death, congestive heart failure (CHF) and cerebral ischemic stroke. The results of CAG were interpreted by two experienced cardiologists.

### 2.2 Exclusion criteria

Patients with any of the following conditions were excluded: occlusion or subtotal occlusion of the right coronary artery (RCA) and left anterior descending artery (LAD); missing significant including baseline ECGs at the onset of the disease and the time of emergency presentation or ward admission and PCI; left/right bundle branch block, intraventricular delay, or pacemaker‐dependent rhythm on the ECG. Other exclusion criteria included: a history of coronary artery bypass grafting, pacemaker implantation, and tumors; severe liver or kidney insufficiency, rheumatic heart disease, cardiomyopathy, myocarditis, congenital heart disease, blood system disease, and acute infection.

### 2.3 Definition of “N” wave

**Figure 1:**
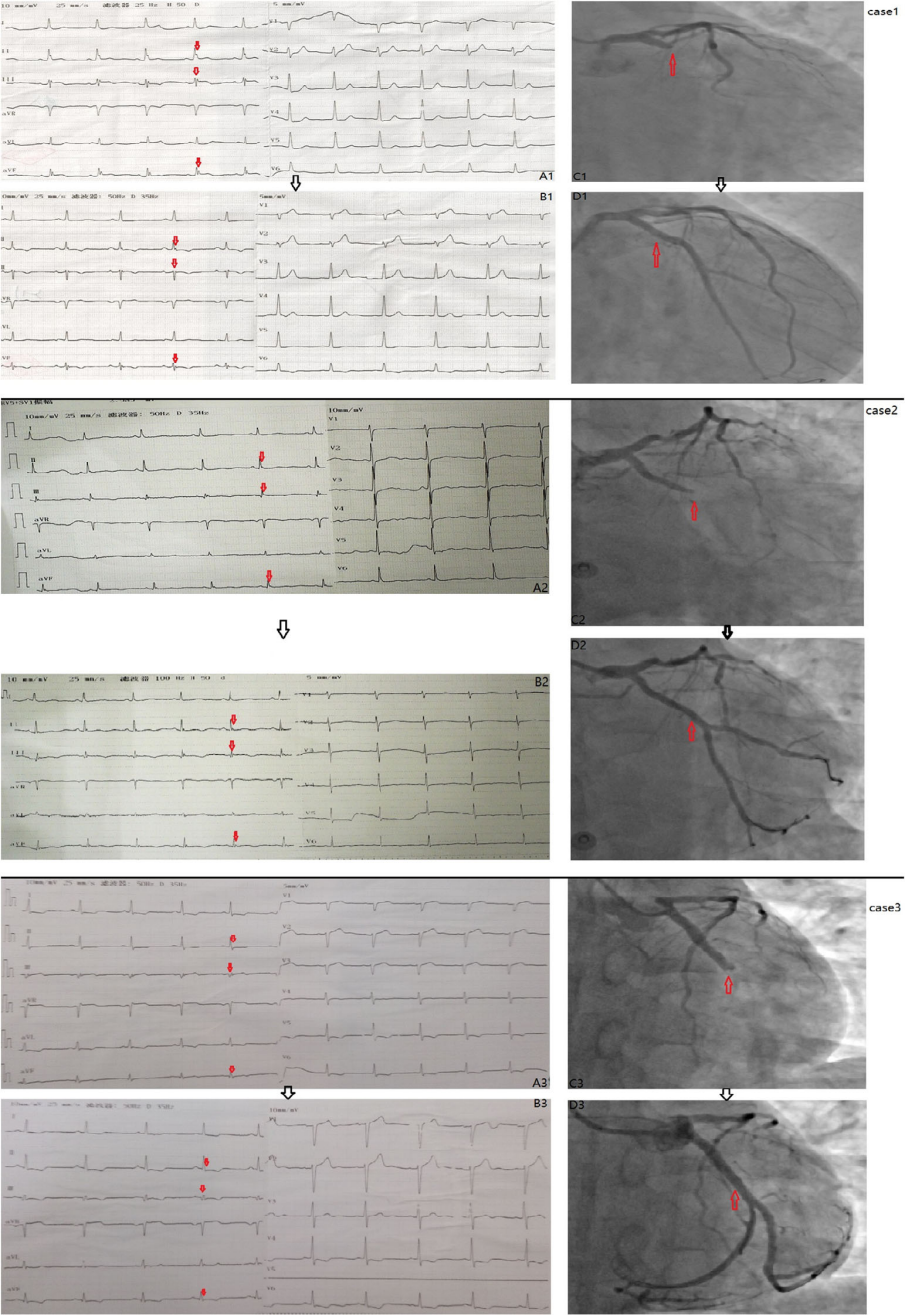
The electrocardiograms and the coronary angiograms before and after PCI of three N‐NSTEMI cases. “N” wave and the electrocardiographic evolution of “N” wave myocardial infarction: A notch or deflection was present in the terminal QRS complex (red arrow, A1, A2, A3); The notch or deflection in the terminal QRS complex of the same patient changed 2 hours later (red arrow, B1, B2, B3). Coronary angiography: Demonstrated acute LCX occlusion of the same patient before PCI (red arrow, C1, C2, C3) and demonstrated LCX artery re‐opened after PCI (red arrow, D1, D2, D3)

According to the following criteria, a “N” wave was considered present in the ECG:^1^ (a) a notch or deflection in the terminal QRS complex of the surface ECG (Figure 1, red arrow, A1, A2, A3); (b) the height of notch or deflection of ≥2 mm (the point of deflection was measured with reference to the PR segment); (c) a continuous change of the notch (the point of deflection shifted ≥2 mm with reference to the PR segment, ≥2 leads) in 24 hours, even disappeared or come into the “s” wave (Figure 1, red arrow, B1, B2, B3); (d) with a prolongation of QRS wave duration in these leads. We list the electrocardiograms and coronary angiograms of three N‐NSTEMI cases in Figure 1, the results of coronary angiography corresponding to ECG before PCI are shown in Figure 1 (red arrow, C1, C2, C3), and the coronary angiography results of these three patients after PCI are shown in Figure 1 (red arrow, D1, D2, D3). The above ECG criteria and coronary angiographic results were judged by two cardiologists, who jointly reconciled differences or consulted a third cardiologist there was any disagreement (Figure 1).

### 2.4 Grouping and Follow up

Grouping: According to whether there were two or more lead ST elevations in 18 lead ECG, 322 patients with STEMI and 552 patients with NSTEMI were enrolled. Based on the presence of “N” wave in the ECG, patients with NSTEMI were further categorized as having either “N” wave (N‐NSTEMI group, n = 232) or non‐N‐wave (non N‐NSTEMI group, n = 320).

Outcome variables: All patients were followed up clinically by telephone contact or office visit. MACEs were defined as cardiogenic shock, no‐reflow, ST, TVR, TLR, rUAP, all‐cause death, chronic heart failure, and cerebral ischemic stroke.^3^, ^4^ Angiographic success was defined as a final residual stenosis of <30% with TIMI grade 3 flow or improvement in flow as compared with the diagnostic angiogram. ST was recognized by angiographic evidence of partial or total stent occlusion with or without the presence of thrombus. No reflow refers to the reopening of severely narrowed or obstructed vessels to restore blood flow, but the ischemic area is not adequately perfused. TVR was defined as a revascularization in any lesion in the index vessel and TLR was defined as a repeat revascularization in the stent or in 5‐mm segments on the either side of the stent.

### 2.5 Statistical analysis

Continuous variables were expressed as mean ± SD, and *t* tests were used for comparison between two groups. Categorical variables were presented as counts and percentage (%). Their inter‐group comparisons were conducted using *x* ^2^ tests. MACE was determined with the Kaplan‐Meier curve. Logistic multivariate analysis was used to analyze the relationship between N‐NSTEMI, STEMI, and MACE during hospitalization. A *P* value <.05 was considered as statistically significant. All statistical analyses were performed using the SPSS 22.0 software.

## 3 RESULTS

### 3.1 Comparison of baseline characteristics

Baseline demographic and clinical characteristics were compared among three groups, (Table 1). In STEMI and N‐NSTEMI groups, myocardial infarction markers were significantly higher than those in the non N‐NSTEMI group (*P* < .001), but they were no significant difference between N‐NSTEMI and STEMI groups (Table 1). The LVEF, LVEDD and the rate of LVEF <40% were similar in STEMI and N‐NSTEMI groups, which was significantly lower than the non N‐NSTEMI group (*P* < .05; Table 1).

**Table 1:** Patients' baseline demographic and clinical characteristic by study groups [*x* ± *s*, n (%)]

		NSTEMI		
	STEMI (n = 322)	N‐NSTEMI (n = 232)	non N‐NSTEMI (n = 320)	*P* value (all groups)
**General clinical information**				
Male (n, %)	175 (54.3%)	122 (52.6%)	162 (50.6%)	.174
Age (year)	60.7 ± 10.4	62.8 ± 10.4	63.7 ± 9.1	.109
BMI (kg/m^2^)	23.5 ± 3.4	23.8 ± 3.6	22.9 ± 3.1	.288
**Personal history**				
Smoking (n, %)	157 (48.8%)	109 (47.0%)	126 (39.4%)	.000
Hypertension (n, %)	158 (49.1%)	166 (71.6%)	245 (76.6%)	.000
Diabetes (n, %)	144 (44.7%)	86 (37.1%)	141 (44.1%)	.000
Hyperlipidemia (n, %)	132 (41.0%)	90 (38.8%)	133 (41.6%)	.122
Myocardial infarction (n, %)	36 (11.2%)	37 (15.9%)	47 (14.7%)	.002
**Family history**				
Myocardial infarction (n, %)	52 (16.1%)	39 (16.8%)	55 (17.2%)	.286
Cerebral infarction (n, %)	11 (3.4%)	7 (3.1%)	9 (2.8%)	.297
**Biochemical indices**				
CHOL (mmol/L)	4.2 ± 1.8	4.3 ± 1.8	4.2 ± 1.6	.381
TG (mmol/L)	2.1 ± 0.9	2.0 ± 0.9	1.9 ± 0.9	.087
LDL (mmol/L)	3.0 ± 1.2	3.1 ± 1.3	3.0 ± 1.2	.312
HbA1C (%)	7.9 ± 3.6	7.6 ± 3.8	7.7 ± 3.2	.071
FPG (mmol/L)	7.1 ± 2.8	7.1 ± 3.0	7.1 ± 2.9	.451
ALT (U/L)	32.7 ± 12.4	33.1 ± 13.8	33.2 ± 12.9	.299
BNP (pg/mL)	175.3 ± 86.4	176.5 ± 88.1	174.6 ± 90.3	.284
CR (umol/L)	79.1 ± 12.6	78.6 ± 11.9	79.2 ± 11.9	.321
**Myocardial infarction marker (max)**				
AST (U/L)	126.6 ± 45.2	128.2 ± 50.1	62.6 ± 29.1	.000
CK (U/L)	756.4 ± 98.1	764.5 ± 91.8	301.6 ± 89.2	.000
CK‐MB (U/L)	96.4 ± 22.2	91.5 ± 24.1	24.0 ± 6.1	.000
TnI (μg/L)	42.4 ± 21.8	37.4 ± 20.1	9.5 ± 5.2	.000
**Drug treatment**				
Aspirin (n, %)	322 (100%)	232 (100%)	320 (100%)	‐
Clopidogrel (n, %)	185 (57.5%)	132 (56.9%)	190 (59.4%)	.039
Ticagrelor (n, %)	137 (42.5%)	100 (43.1%)	130 (40.6%)	.144
Low molecular weight heparin (n, %)	301 (93.5%)	215 (92.7%)	303 (94.7%)	.192
Statins (n, %)	322 (100%)	232 (100%)	320 (100%)	‐
β‐receptor blocker (n, %)	165 (51.2%)	123 (53.0%)	162 (50.6%)	.274
ACEIs/ARBs (n, %)	104 (32.3%)	76 (32.8%)	110 (34.4%)	.196
**Echocardiographic data**				
LVEF (%)	55.4 ± 7.2	56.4 ± 7.0	60.5 ± 6.9	.000
LVEF <40% (n, %)	24 (7.5%)	15 (6.5%)	11 (3.4%)	.001
LVEDD (mm)	50.7 ± 8.7	50.9 ± 9.0	47.5 ± 8.5	.001
**PCI information**				
Complete occlusion of LCX (n, %)	283 (87.9%)	196 (84.5%)	94 (29.4%)	.000
Right coronary dominance pattern (n, %)	75 (23.3%)	59 (25.4%)	126 (39.4%)	.000
Mean diameter of stent (mm)	2.8 ± 0.7	2.8 ± 0.8	2.9 ± 0.7	.101
Mean length of stent (mm)	28.6 ± 9.1	29.3 ± 9.6	28.4 ± 10.1	.183
Thrombus aspiration (n, %)	9 (2.8%)	2 (0.8%)	0	.000
IABP (n, %)	2 (0.6%)	1 (0.4%)	0	.043
No‐reflow (n, %)	5 (1.5%)	19 (8.2%)	5 (1.6%)	.000
**Door‐to‐balloon time (n, %)**				
<6 hours	295 (91.6%)	54 (23.3%)	61 (19.1%)	.000
6 hours to 12 hours	15 (4.7%)	50 (21.6%)	62 (19.4%)	.000
>12 hours	12 (3.7%)	128 (55.2%)	197 (61.5%)	.000
**Location of culprit lesion (n, %)**				
proximal LCX before giving rise to OM1	255 (79.2%)	198 (85.2%)	57 (17.8%)	.000
distal LCX after giving rise to OM1	49 (15.2%)	26 (11.2%)	201 (62.8%)	.000
OM1	18 (5.6%)	8 (3.5%)	62 (19.4%)	.000

Abbreviations: ACEIs, angiotension converting enzyme inhibitors; ARBs, Ang II receptor blocker; ALT, alanine aminotransferase; AST, glutamic oxaloacetic transaminase; BMI, body mass index; BNP, brain natriuretic peptide; CHOL, total cholesterol; CR, creatinine; CK, creatine kinase; CK‐MB, creatine kinase isoenzyme MB; FPG, fasting blood glucose; IABP, intra‐aortic balloon pumps; LCX, left circumflex artery; LDL, low density lipoprotein; LVEDD, left ventricular end diastolic diameter; LVEF, left ventricular ejection fraction; N‐NSTEMI, “N” wave non‐ST‐elevated myocardial infarction; non N‐NSTEMI, non‐“N”‐wave non‐ST‐elevated myocardial infarction; PCI, percutaneous coronary intervention; STEMI, ST‐elevated myocardial infarction; TG, triglyceride; TnI, troponin I.

The information of PCI was compared among study groups (Table 1). We found that the incidence rate of no‐reflow was higher in the N‐NSTEMI group than in the STEMI and non N‐NSTEMI groups (N‐NSTEMI: 8.2% vs STEMI: 1.5% vs non N‐NSTEMI: 1.6%, *P* < .001). The rate of complete coronary occlusion in the N‐NSTEMI and STEMI groups were higher than in the non N‐NSTEMI group (STEMI: 87.9% vs N‐NSTEMI: 84.5% vs non N‐NSTEMI: 29.4%, *P* < .001). In the STEMI and N‐NSTEMI groups, the proportion of the right dominant type was similar, which was significantly lower than the non N‐NSTEMI group (STEMI: 23.3% vs N‐NSTEMI: 25.4% vs non N‐NSTEMI: 39.4%, *P* < .001). The percentage of patients with thrombus aspiration in the STEMI group was significantly greater than in N‐NSTEMI and non N‐NSTEMI groups (STEMI: 2.8% vs N‐NSTEMI: 0.8% vs non N‐NSTEMI: 0, *P* < .001; Table 1).

The operation time was compared among study groups (Table 1). The proportion of patients in the STEMI group with time‐to‐operation (door‐to‐balloon time) <6 hours was significantly greater than that in N‐NSTEMI and non N‐NSTEMI groups (STEMI: 91.6% vs N‐NSTEMI: 23.3% vs non N‐NSTEMI: 19.1%, *P* < .001), the proportion of patients with a time‐to‐operation of 6 to 12 hours and >12 hours were significantly greater in the non N‐NSTEMI and N‐NSTEMI group than its in the STEMI group (6‐12 hours: STEMI: 4.7% vs N‐NSTEMI: 21.6% vs non N‐NSTEMI: 19.4%, *P* < .001; >12 hours: STEMI: 3.7% vs N‐NSTEMI: 55.2% vs non N‐NSTEMI: 61.5%, *P* < .001), and it did not differ significantly between the former two groups (Table 1).

The lesion location was compared among all patients (Table 1). The lesions in the N‐NSTEMI and STEMI groups were often located proximal LCX before giving rise to OM1 of LCX artery; however, the non N‐NSTEMI group was often located distal LCX after giving rise to OM1 and the OM1 (proximal LCX before giving rise to OM1:STEMI: 79.2% vs N‐NSTEMI: 85.2% vs non N‐NSTEMI: 17.8%, *P* < .001; distal LCX after giving rise to OM1: STEMI: 15.2% vs N‐NSTEMI: 11.2% vs non N‐NSTEMI: 62.8%, *P* < .001; the OM1: STEMI: 5.6% vs N‐NSTEMI: 3.5% vs non N‐NSTEMI: 19.4%, *P* < .001; Table 1).

### 3.2 Comparison of MACEs in hospital and 12‐months

The occurrence of MACEs was compared among the study groups (Table 2). We found that the incidence rate of all MACEs in N‐NSTEMI and STEMI groups was significantly higher than those in the non N‐NSTEMI group (STEMI: 11.2% vs N‐NSTEMI: 11.6% vs non N‐NSTEMI: 2.8%, *P* < .001), but it was similar between N‐NSTEMI and STEMI groups (*P* = .85). During hospitalization, the incidence rate of all‐cause death in N‐NSTEMI and STEMI groups was significantly higher than those in the non N‐NSTEMI group (STEMI: 1.2% vs N‐NSTEMI: 1.2% vs non N‐NSTEMI: 0.3%, *P* < .05). At 12 months, the incidence rates of all‐cause death, ST, TVR, and recurrent UAP in N‐NSTEMI and STEMI groups were higher than those in the non N‐NSTEMI group (all‐cause death: STEMI: 1.2% vs N‐NSTEMI: 1.3% vs non N‐NSTEMI: 0.3%, *P* < .05; ST: STEMI: 0.9% vs N‐NSTEMI: 0.9% vs non N‐NSTEMI: 0, *P* < .05; TVR: STEMI: 1.6% vs N‐NSTEMI: 1.8% vs non N‐NSTEMI: 0.3%, *P* < .001; recurrent UAP: STEMI: 1.2% vs N‐NSTEMI: 1.8% vs non N‐NSTEMI: 0.3%, *P* < .001).

**Table 2:** Hospitalization period and 12‐months major cardiac adverse event rate

		NSTEMI		
	STEMI (n = 322)	N‐NSTEMI (n = 232)	non N‐NSTEMI (n = 320)	*P* value (all groups)
**All MACEs**	36 (11.2%)	27 (11.6%)	9 (2.8%)	.000
**In‐hospital MACEs (n, %)**				
Number of patients (n)	322	232	320	
ST	1 (0.3%)	1 (0.4%)	0	.087
TVR	1 (0.3%)	1 (0.4%)	0	.087
TLR	0	0	0	‐
All‐cause death	4 (1.2%)	3 (1.2%)	1 (0.3%)	.003
Cardiogenic shock	3 (0.9%)	2 (0.8%)	1 (0.3%)	.057
Congestive heart failure	2 (0.6%)	2 (0.8%)	1 (0.3%)	.081
Cerebral ischemic stroke	0	0	0	‐
**12‐Months MACEs (n, %)**				
Number of patients (n)	315	230	310	
ST	3 (0.9%)	2 (0.9%)	0	.002
TVR	5 (1.6%)	4 (1.8%)	1 (0.3%)	.000
TLR	3 (0.9%)	3 (1.3%)	2 (0.6%)	.145
All‐cause death	4 (1.2%)	3 (1.3%)	1 (0.3%)	.001
Recurrent UAP	4 (1.2%)	4 (1.8%)	1 (0.3%)	.000
Congestive heart failure	3 (0.9%)	2 (0.9%)	1 (0.3%)	.072
Cerebral ischemic stroke	1 (0.3%)	0	0	.155

Abbreviations: MACE, major adverse cardiac events; N‐NSTEMI, “N” wave non‐ST‐elevated myocardial infarction; non N‐NSTEMI, non‐“N”‐wave non‐ST‐elevated myocardial infarction; STEMI, ST‐elevated myocardial infarction, ST, stent thrombosis; TVR, target vessel revascularization; TLR, target lesion revascularization; UAP, unstable angina pectoris.

**Figure 2:**
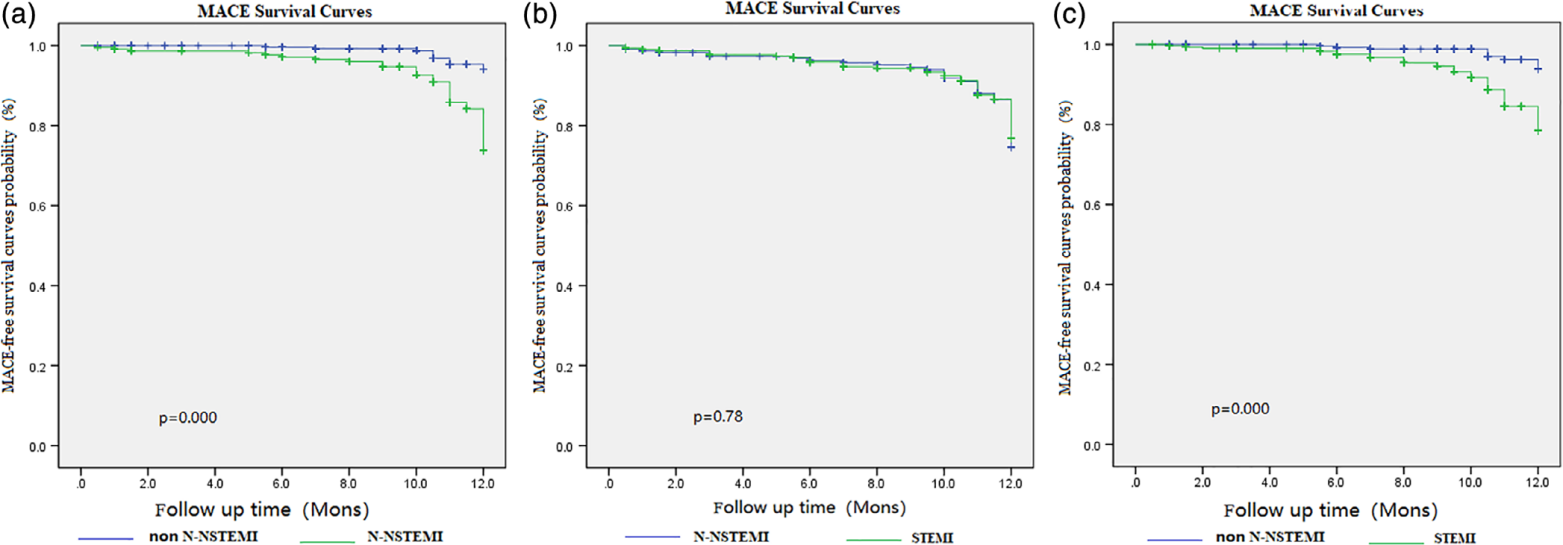
The Kaplan‐Meier graph of MACE‐free survival. The MACE‐free survival were comparable between N‐NSTEMI and STEMI groups (*P* = .78; B). But it was significant difference between N‐NSTEMI and non N‐NSTEMI groups (*P* = .000; A), and between STEMI and non N‐NSTEMI groups (*P* = .000; C). MACE, major adverse cardiac events; N‐NSTEMI, “N” wave non‐ST‐elevated myocardial infarction; non N‐NSTEMI, non‐“N”‐wave non‐ST‐elevated myocardial infarction; STEMI, ST‐elevated myocardial infarction

The Kaplan‐Meier analysis showed that the MACE‐free survival were comparable between N‐NSTEMI and STEMI groups (*P* = .78; Figure 2B). But it was significant difference between N‐NSTEMI and non N‐NSTEMI groups (*P* < .001; Figure 2A), and between STEMI and non N‐NSTEMI groups (*P* < .001; Figure 2C).

### 3.3 Logistic multivariate analysis

**Figure 3:**
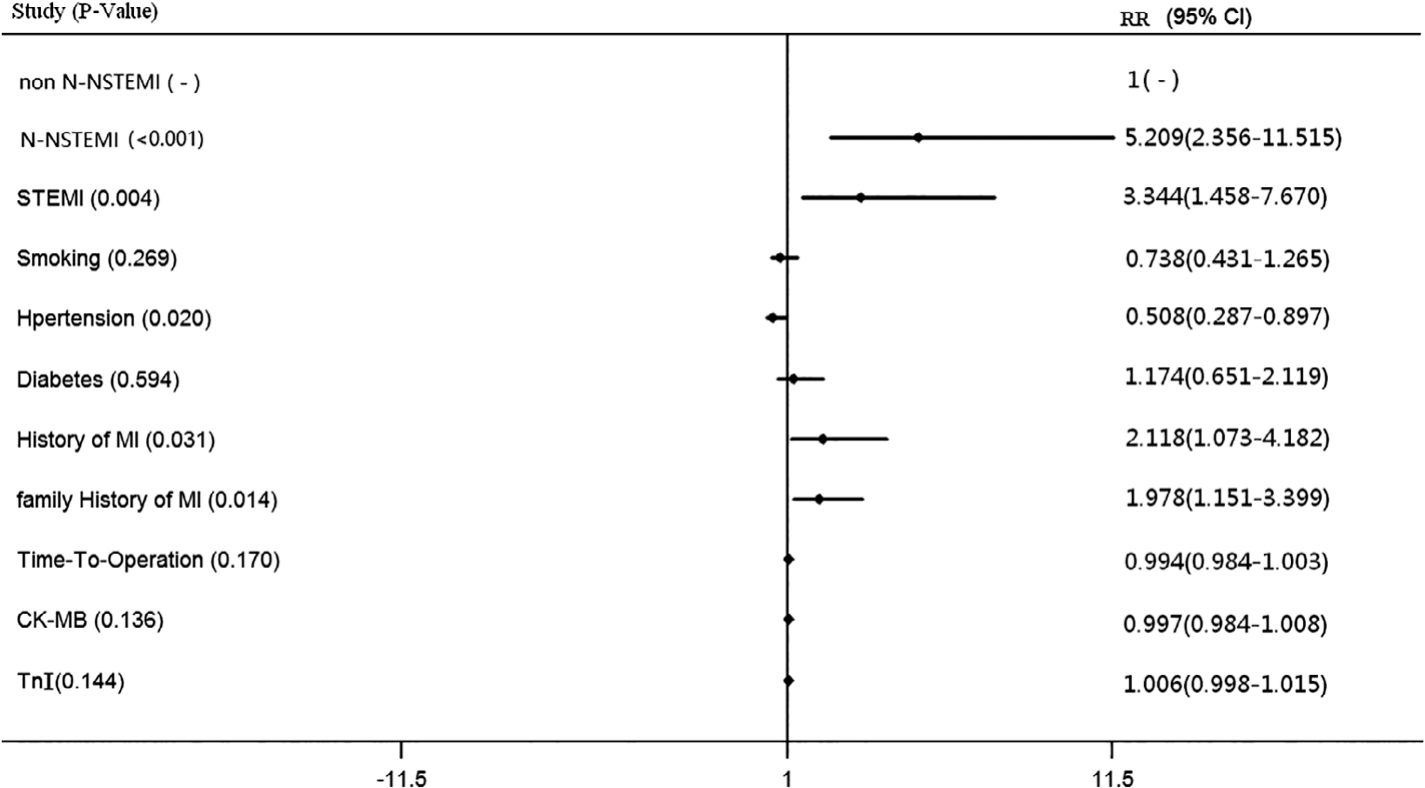
Logistic multivariate analysis. Adjusted for non N‐NSTEMI, smoking, hypertension, diabetes, history of MI, family history of MI, time‐to‐operation, CK‐MB and TnI, both N‐NSTEMI and STEMI are independent risk factors for MACE during hospitalization. CK‐MB, creatine kinase isoenzyme MB; MI, myocardial infarction; N‐NSTEMI, “N” wave non‐ST‐elevated myocardial infarction; non N‐NSTEMI, non‐“N”‐wave non‐ST‐elevated myocardial infarction; STEMI, ST‐elevated myocardial infarction; TnI, troponin I

Logistic multivariate analysis was used to analyze the relationship between N‐NSTEMI, STEMI, and MACE during hospitalization and 12 months (Figure 3). Adjusted for non N‐NSTEMI, smoking, hypertension, diabetes, history of MI, family history of MI, time‐to‐operation, CK‐MB, and TnI, both N‐NSTEMI and STEMI are independent risk factors for MACE (N‐NSTEMI: 5.209 (2.356‐11.515), *P* < .001; STEMI: 3.344 (1.458‐7.670), *P* = .004).

## 4 DISCUSSION

The detection sensitivity is very low (approximately 32%~50%) for acute occlusion in the LCX, electrocardiogram was mostly NSTEMI.^1^, ^4^, ^5^, ^6^, ^7^ This may be because LCX is located on the posterolateral side of the heart, mainly supplying the lateral and posterior walls of the basal part of the left ventricle, far from the chest wall, and lacking suitable leads for electrocardiogram, called the “blind area.”^1^, ^8^, ^9^, ^10^, ^11^ Niu et al^1^ were the first to report delayed activation wave (“N” wave) in NSTEMI patients with culprit vessel in LCX. Angiography showed that in NSTEMI patients with “N” wave on electrocardiogram, 77% of the culprit vessels were in the circumflex artery, 6% were in the left anterior descending and 18% were in the right coronary artery, and the sensitivity and specificity of “N” wave for detection the left circumflex artery lesions were 77% and 96%. Previously, the clinical features of AMI patients with an “N” wave (delayed activation wave) in their electrocardiogram were not clear, and the clinical characteristic of AMI patients with “N” wave, STEMI patients and patients with non‐ “N” wave had not been studied. Therefore, in this article, we analyzed the clinical data of patients with AMI and culprit vessel in left circumflex artery, and explored the similarities and differences between acute NSTEMI with “N” wave in ECG and acute STEMI.

There were some significant findings in this study after analyzing the clinical feature of AMI and culprit vessel in left circumflex artery. First, we found that the myocardial infarction markers (AST, TnI, CK, and CK‐MB) were greater in STEMI and N‐NSTEMI groups than in the non N‐NSTEMI group, which indicated a larger area of myocardial ischemia and or infarction. Greater ischemia in STEMI and N‐NSTEMI groups occurred because of the higher incidence of acute occlusion in LCX artery, but there was no difference between these two groups. Second, the lesions in the N‐NSTEMI and STEMI groups were often located proximal LCX before giving rise to OM1 of LCX artery, however the non N‐NSTEMI group was often located distal LCX after giving rise to OM1 and the OM1, and the rate of complete coronary occlusion in the N‐NSTEMI and STEMI groups were higher than in the non N‐NSTEMI group. This is probably because rapid conducting cardiac Purkinje fibers are mostly located in the lower left ventricle. The distribution of Purkinje's network is situated mainly in the lower half of the ventricles and is virtually absent in basal regions, therefore, these fibers cannot supply an electrical contribution either in normal condition or in the presence of limited damage. This endocardium constitutes a histological‐functional entity, since the Purkinje fibers are depolarized simultaneously without producing differences in potential.^1^, ^2^, ^11^ A series of abnormal electrical activities occur due to prolonged ischemia that results in asynchronous depolarization of the corresponding ventricular muscle, and then the delayed activation wave is formed. Consequently, a specific notch appears at the terminal part of the QRS complex‐“N” wave is seen on ECG, which evolves dynamically with restoration of localized blood supply.^1^, ^12^, ^13^ Therefore, the delayed activation wave is a new pattern of ischemia in ECG, N‐wave can be regarded as a manifestation of LCX proximal stenosis or even complete occlusion, which is equivalent to ST‐segment elevation. However, greater detail about this mechanism still needs to be clarified in further studies. In addition, the proportion of right coronary dominant type between STEMI group and N‐NSTEMI group was similar, but significantly lower than that in non N‐NSTEMI group. It could be because that size of the coronary artery to the myocardium is related to the size of the coronary artery, the number of branches, and the location of the distribution.^1^, ^14^ When the acute lesion is located in the proximal part of LCX, it will cause a greater myocardial damage and necrosis that can result in extensive ST segment changes in ECG. In some patients, it can cause significant ST segment elevation when the coronary artery is completely occluded, or in some other patients, it can behave as delayed activation wave. Therefore, in STEMI and N‐NSTEMI groups, most of the criminal lesions were located in the proximal segment of LCX and the rate of complete coronary occlusion is higher.

The incidence rate of no‐reflow events was significantly higher in the N‐NSTEMI group than in STEMI and non N‐NSTEMI group. Possible reason for this difference was that emergency vascularization was not performed as early as possible within 12 hours after onset and CAG and stent implantation were implemented at a later stage as N‐NSTEMI was considered as a category of NSTEMI.^1^, ^15^ As a result, the no‐reflow frequently occurred during this aforementioned critical time period. For most STEMI patients, vascularization was implemented earlier after onset, so the no‐reflow rate was low. But surprisingly, the incidence rate of all MACEs and all‐cause death in N‐NSTEMI and STEMI groups were significantly higher than those in the non N‐NSTEMI group, but they were similar between N‐NSTEMI and STEMI groups. Moreover, at 12 months, the incidence rates of ST, TVR, recurrent unstable angina pectoris in N‐NSTEMI and STEMI groups were higher than those in the non N‐NSTEMI group. Thus, further follow‐up confirmed that the incidence rate of MACEs in NSTEMI and N‐NSTEMI patients was similar after PCI, it was higher than that in non N‐NSTEMI patients. It possibly because the area of myocardial infarction was larger as the culprit lesion was more often located at the proximal segments of LCX and its stenosis was more severe, this also confirms our previous conclusions.^16^, ^17^ Logistic multivariate analysis found that both N‐NSTEMI and STEMI are independent risk factors for MACE, this reflects N‐NSTEMI has a risk equivalent to acute STEMI. Some studies also suggested that the “N” wave resulted from a greater dispersion degree of transmural repolarization, in which the electrocardiographic activity was unstable to induce malignant ventricular arrhythmia and thus increased the risk for ventricular tachycardia, ventricular fibrillation, or sudden death.^18^, ^19^, ^20^, ^21^ In a recent meta‐analysis, the NSTEMI patients with complete occlusion of culprit vessel detected by CAG were at a higher risk of all‐cause death and MACEs, which was similar whit STEMI.^22^ Study found that 27% of NSTEMI patients had an occluded infarct artery and the lesions were more frequently located in arteries supplying the inferolateral myocardium and were associated with larger infarct sizes and higher mortality.^9^

NSTEMI patients with “N” wave in ECG that the LCX was the infarct‐related artery had a higher incidence of acute occlusion and MACEs than non N‐NSTEMI patients, they did not always have a more favorable clinical outcome than patients with acute LAD or RCA occlusion.^1^, ^12^ Therefore, N‐NSTEMI has a risk equivalent to acute STEMI, these NSTEMI patients with the delayed activation wave in which classic ST‐segment elevation is absent, may benefit from earlier revascularization.

## 5 CONCLUSION

In AMI with the culprit vessel in LCX, the lesion sites, the stenosis severity, and the incidence rate of MACEs during hospitalization and at 12‐months were similar in N‐NSTEMI and STEMI patients, both N‐NSTEMI and STEMI are independent risk factors for MACE; “N” wave NSTEMI has a risk equivalent to acute STEMI.

## 6 LIMITATION

The present research was a single‐center study with limited range and number of cases; therefore, it may be subject to selection bias. In the future, a multi‐center, randomized, prospective, large sample‐size study needs to be conducted to further explore the clinical features of AMI patients with “N” wave.

## CONFLICT OF INTEREST

The authors declare no potential conflict of interest.

## AUTHORS CONTRIBUTIONS

T.Y., J.C., and X.L. wrote the manuscript and researched the data. C.X. and T.N. contributed to discussion. P.F. and X.F. designed the study and reviewed the data and revised the manuscript. All authors read and approved the final manuscript.

## ETHICS STATEMENT

This study was approved by the Ethics Committee of Shengjing Hospital. Written informed consents were obtained from all enrolled AMI inpatients.

## Data Availability

All data generated or analyzed during this study are included in this article.

## References

[1] Niu T , Fu P , Jia C , et al. The delayed activation wave in non‐ST‐elevation myocardial infarction. Int J Cardiol. 2013;162(2):107‐111. 21663984 10.1016/j.ijcard.2011.05.063

[2] Das MK , Suradi H , Maskoun W , et al. Fragmented wide QRS on a 12‐lead ECG: a sign of myocardial scar and poor prognosis. Circ Arrhythm Electrophysiol. 2008;1(4):258‐268. 19808417 10.1161/CIRCEP.107.763284

[3] Kadi H , Demir AK , Ceyhan K , et al. Association of fragmented QRS complexes on ECG with left ventricular diastolic function in hypertensive patients. Turk Kardiyol Dern Ars. 2015;43(2):149‐156. 25782119 10.5543/tkda.2015.04495

[4] Çanga A , Kocaman SA . Relationship between fragmented QRS complexes and left ventricular systolic and diastolic functions. Herz. 2013;38(6):665‐670. 23588600 10.1007/s00059-012-3739-1

[5] Schmitt C , Lehmann G , Schmieder S , et al. Diagnosis of acute myocardial infarction in angiographically documented occluded infarct vessel. Chest. 2001;120:1540‐1546. 11713132 10.1378/chest.120.5.1540

[6] Berry C , Zalewski A , Kovach R , Savage M , Goldberg S . Surface electrocardiogram in the detection of transmural myocardial ischemia during coronary artery occlusion. Am J Cardiol. 1989;63:21‐26. 2521194 10.1016/0002-9149(89)91069-2

[7] Huey BL , Beller GA , Kaiser DL , Gibson RS . A comprehensive analysis of myocardial infarction due to left circumflex artery occlusion: comparison with infarction due to right coronary artery and left anterior descending artery occlusion. J Am Coll Cardiol. 1988;12:1156‐1166. 3170958 10.1016/0735-1097(88)92594-6

[8] Schweitzer P . The electrocardiographic diagnosis of acute myocardial infarction in the thrombolyticera. Am Heart J. 1990;119:642‐654. 2178372 10.1016/s0002-8703(05)80288-1

[9] Wang TY , Zhang M , Fu Y , et al. Incidence, distribution, and prognostic impact of occluded culprit arteries among patients with non–ST‐elevation acute coronary syndromes undergoing diagnostic angiography. Am Heart J. 2009;157:716‐723. 19332201 10.1016/j.ahj.2009.01.004

[10] Dixon WC , Wang TY , Dai D , et al. Anatomic distribution of the culprit lesion in patients with non–ST‐segment elevation myocardial infarction undergoing percutaneous coronary intervention: findings from the National Cardiovascular Data Registry. J Am Coll Cardiol. 2008;52:1347‐1351. 18929247 10.1016/j.jacc.2008.07.029

[11] Stribling WK , Kontos MC . AbbateA, et al. left circumflex occlusion in acute myocardial infarction (from the National Cardiovascular Data Registry). Am J Cardiol. 2011;108:959‐963. 21820644 10.1016/j.amjcard.2011.05.027

[12] Haisaguerre M , Derval N , Sacher F , et al. Sudden cardiac arrest a ssociated with early repolarization. N Engl J Med. 2008;358:2016‐2023. 18463377 10.1056/NEJMoa071968

[13] Yan GX , Anzelevitch C . Cellular basis for the electrocardiographic J wave. Circulation. 1996;93:372‐378. 8548912 10.1161/01.cir.93.2.372

[14] Maruyama M , Atarashi H , Ino T , Kishida H . Osborn waves associated with ventricular fibrillation in a patient with vasospastic angina. J Cardiovasc Electrophysiol. 2002;13:486‐489. 12030532 10.1046/j.1540-8167.2002.00486.x

[15] Rituparna S , Suresh S , Chandrasshekhar M , et al. Occurrence of “J waves” in 12 lead ECG as a marker of acute ischemia and their cellular basis. Pacing Clin Electrophysiol. 2007;30:817‐819. 17547622 10.1111/j.1540-8159.2007.00760.x PMC1989774

[16] Jastrzebski M , Kukla P . Ischemic J wave: novel risk marker for ventricular fibrillation. Heart Rhythm. 2009;6:829‐835. 19467513 10.1016/j.hrthm.2009.02.036

[17] Bauer T , Gitt AK , Hochadel M . Left circumflex arter‐related myocardial infarction: dose ST elevation matter? Results from the Euro Heart Survey PCI registry. Int J Cardiol. 2013;168(6):5239‐5242. 23998547 10.1016/j.ijcard.2013.08.024

[18] Choi JH , Chang SA , Choi JO , et al. Frequency of myocardial infarction and its relationship to angiographic collateral flow in territories supplied by chronically occluded coronary arteries. Circulation. 2013;127(6):703‐709. 23277308 10.1161/CIRCULATIONAHA.112.092353

[19] Missiri AME , Guindy RR . Echocardiographic assessment of right ventricular functions in patients with proximal right coronary artery chronic total occlusion. Int J Cardiovasc Imaging. 2016;32(6):895‐903. 26850438 10.1007/s10554-016-0850-z

[20] Gecmen C , Candan O , Kahyaoglu M , et al. Echocardiographic assessment of right ventricle free wall strain for prediction of right coronary artery proximal lesion in patients with inferior myocardial infarction. Int J Cardiovasc Imag. 2018;34(4):1‐8. 10.1007/s10554-018-1325-1 29479662

[21] Bakhoum SWG , Sorour SM , Elramly MZ , et al. Impact of waist circumference on hospital outcome and coronary angiographic findings of patients with acute ST‐segment elevation myocardial infarction. Egypt Heart J. 2015;67(2):159‐165.

[22] Khan AR , Golwala H , Tripathi A , et al. Impact of total occlusion of culprit artery in acute non‐ST elevation myocardial infarction: a systematic review and meta‐analysis. Eur Heart J. 2017;38(41):3082‐3089. 29020244 10.1093/eurheartj/ehx418

